# Fungal microbiome in inflammatory bowel disease: a critical assessment

**DOI:** 10.1172/JCI155786

**Published:** 2022-03-01

**Authors:** David M. Underhill, Jonathan Braun

**Affiliations:** 1 F. Widjaja Inflammatory Bowel and Immunobiology Research Institute,; 2 Division of Gastroenterology, Department of Medicine, and; 3 Research Division of Immunology, Department of Biomedical Sciences; Cedars-Sinai Medical Center, Los Angeles, California, USA

## Abstract

The gut microbiome is at the center of inflammatory bowel disease (IBD) pathogenesis and disease activity. While this has mainly been studied in the context of the bacterial microbiome, recent advances have provided tools for the study of host genetics and metagenomics of host-fungal interaction. Through these tools, strong evidence has emerged linking certain fungal taxa, such as *Candida* and *Malassezia*, with cellular and molecular pathways of IBD disease biology. Mouse models and human fecal microbial transplant also suggest that some disease-participatory bacteria and fungi may act not via the host directly, but via their fungal-bacterial ecologic interactions. We hope that these insights, and the study design and multi-omics strategies used to develop them, will facilitate the inclusion of the fungal community in basic and translational IBD research.

## Introduction

The influence of the intestinal microbiome in health and disease throughout the body is increasingly being recognized. The potential role of the microbiome in intestinal health and disease is relatively easy to conceptualize, while the effects on peripheral immune responses and diseases such as asthma, heart disease, and cognitive diseases are more challenging to imagine and validate (1). Nevertheless, data continue to mount in animal and human studies supporting the importance of maintaining a microbial population that resists infection and supports immunologic and metabolic homeostasis. Changes in the intestinal microbiome have long been noted in patients with inflammatory bowel disease (IBD), but until very recently, these studies have focused almost exclusively on bacteria in the gut. The intestinal microbiome also includes less well-studied populations of fungi, archaebacteria, and viruses (2, 3). Recent studies have begun to highlight relationships of the fungal mycobiome to various disease states and treatment responses (4–8). This Review aims to investigate our current understanding of the potential for fungi in the gut to contribute to health and disease related to IBD. We will look at features of IBD that suggest a possible role for fungi and antifungal immune responses in the etiology of IBD, at least in a subset of patients.

IBD is the result of a dysregulated inflammatory immune response in the mucosa of the gastrointestinal (GI) tract. Innate and adaptive immune cells drive disease by producing inflammatory mediators that sustain local inflammatory responses and promote expression of adhesion molecules that direct trafficking of further immune cells into the gut. The disease is associated with alterations in the permeability of the intestinal epithelia and in the intestinal microbiota (intestinal “dysbiosis”). IBD is usually classified as Crohn’s disease (CD) or ulcerative colitis (UC) based on phenotypic features of disease location, “patchiness,” and depth of damage to the GI tract.

**Figure 1:**
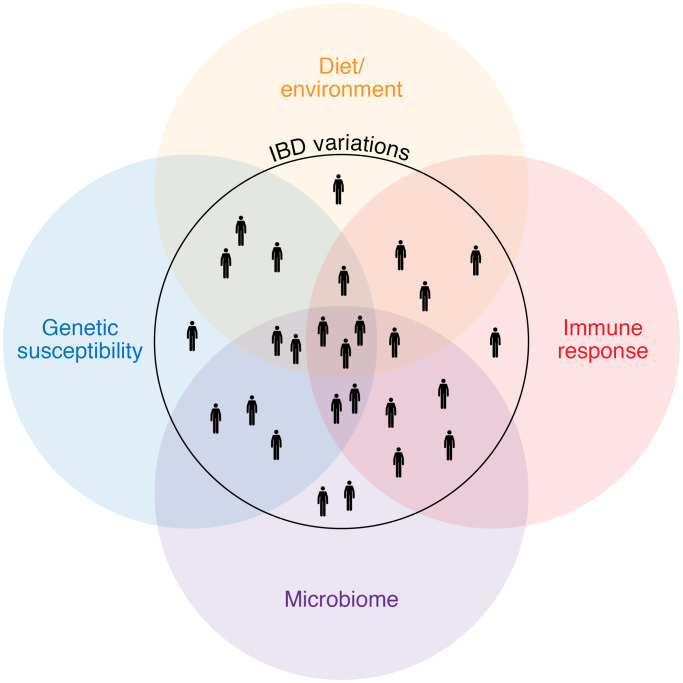
Diversity of IBD presentations and underlying causes. Rather than being a single disease, or two diseases (CD and UC), IBD is increasingly being understood as a multifaceted disorder initiated and exacerbated by influences such as genetic susceptibility and alterations in the intestinal microbiota.

Rather than being a single disease, or two diseases (CD and UC), IBD is increasingly understood as a multifaceted disorder that is initiated and exacerbated by influences such as genetic susceptibility, environmental factors like diet and smoking, differing immune responses, and alterations in the intestinal microbiota (Figure 1). IBD affects an estimated 1.6 million Americans and more than 3.5 million people worldwide. Furthermore, the incidence of IBD is increasing globally (9). A major goal in IBD research and care is to improve outcomes by targeting disease treatment to an individual patient’s specific underlying disease mechanism(s). The heterogeneity of IBD has prompted searches for a marker or combination of markers to distinguish IBD from non-IBD, differentiate IBD subtypes (e.g., UC versus CD), anticipate disease outcomes, and predict response to therapies.

## Genetic and serologic evidence for fungi in IBD

### Genetics.

IBD can affect anyone, although it often runs in families, and it disproportionally affects certain ethnic groups (e.g., Ashkenazi Jews), suggesting some role for genetics in determining risk of developing disease. Genome-wide association studies (GWAS) have implicated over 200 common genetic polymorphisms in contributing to the risk of developing IBD. These associations suggest that specific biologic pathways might be involved in disease, such as innate immunity (e.g., *NOD2*, *CARD9*), mucosal immunity (e.g., *TNFSF15*, *IL23R*), autophagy (e.g., *ATG16L1*, *IRGM*), and epithelial barrier function (e.g., *COL7A1*, *GUCY2C*) (10).

**Figure 2:**
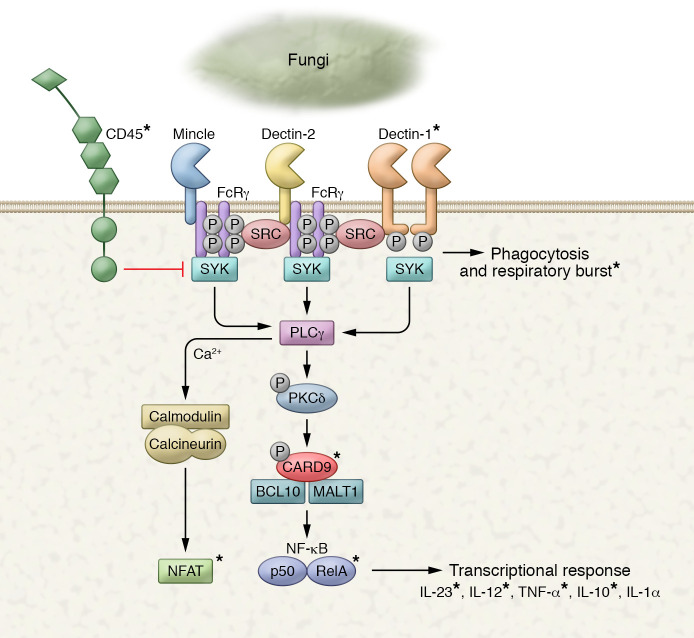
Innate antifungal receptors and key signaling pathways, including through CARD9. Antifungal C-type lectin receptors such as Dectin-1/2 and Mincle have a single extracellular C-type lectin domain. Signaling is mediated either by a short intracellular signaling tail (Dectin-1) or through association in the membrane with the Fc receptor common γ chain (FcRγ) that mediates signaling. Proteins and processes linked to IBD through genetic studies are indicated with asterisks.

For the purposes of the current discussion, the increased risk of developing IBD that is associated with polymorphism in *CARD9* suggests that regulated immune responses to fungi might be important in pathogenesis. Innate immune responses to fungi are mediated in large part by the C-type lectin family of receptors that includes Dectin-1, Dectin-2, and Mincle (2, 11). Upon detecting fungi, these receptors trigger inflammatory responses through a signaling pathway that uses a protein called CARD9 to activate NF-κB (Figure 2). Dectin-1 and CARD9 are understood to be particularly important in antifungal host defense. Deleterious mutations in the genes for both proteins have been specifically associated with recurrent, severe, and sometimes life-threatening fungal infections in humans, and not bacterial or viral infections (12–17).

**Figure 3:**
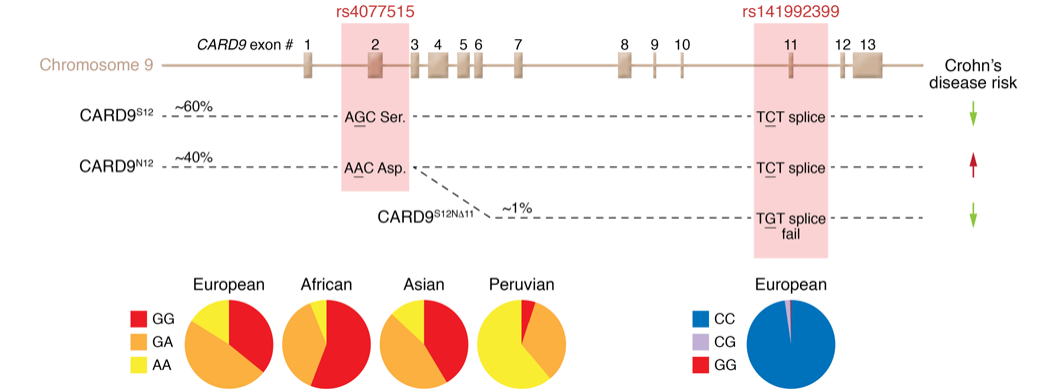
*CARD9* polymorphisms and their effects. The *CARD9* gene comprises 13 exons. A common single-nucleotide polymorphism in exon 2 causes one allele to code for a serine in position 12 and the others to code for an arginine. A second polymorphism at the end of exon 11 results in production of a splice variant lacking exon 11 and production of a protein lacking function. While the N12 variant confers increased risk of developing CD, the truncation variant is protective. (Population distribution from Ensembl [ensembl.org].)

GWAS have linked a nonsynonymous single-nucleotide polymorphism (SNP) in exon 2 of *CARD9* to risk of CD (18–23). This SNP leads to production of a CARD9^S12N^ variant of the protein. The allele frequency of the “minor” N12 coding variant is around 40%, and it is present broadly in all ethnic groups examined (Figure 3). It is slightly less frequent in African populations and slightly more frequent in native South American populations. In some people, a rare second polymorphism at the end of exon 11 results in production of a splice variant lacking exon 11 and production of a protein lacking function (24). While the N12 variant confers increased risk of developing CD, the truncation variant is protective (21–23). The *CARD9^S12N^* polymorphism has also been linked to the risk of developing UC (20), primary sclerosing cholangitis (25), ankylosing spondylitis (26), and IgA nephropathy (27).

The preponderance of data indicates that the primary roles of CARD9 and Dectin-1 (and other CARD9-signaling C-type lectin receptors) are in antifungal immunity, which is consistent with the idea that increased risk of IBD associated with genetic variations in the genes for these proteins is due to alterations in antifungal immunity; however, it is important to note that other potential roles for these genes have been described, largely in mouse studies (28). For example, the mycobacterial cord factor trehalose-6,6-dimycolate (TMD) from *Mycobacterium* *tuberculosis* is potently recognized by the C-type lectin receptor Mincle, which signals through CARD9 to activate proinflammatory responses (29, 30). Mincle has also been implicated in protection against group A *Streptococcus* via recognition of the bacterium’s cell wall lipoteichoic acid monoglucosyldiacylglycerol anchor (31). Parasites including *Schistosoma mansoni* and *Toxoplasma gondii* have been reported to activate inflammatory responses via the CARD9 pathway (32–34). Finally, a role for CARD9 in signaling via intracellular viral RNA and DNA sensors has been postulated involving RIG-I (35) or RAD50 (36). Thus, further studies will be required to clarify whether genes such as *CARD9* are implicated in the risk of IBD due to alterations in antifungal immunity or other effects.

### Antibodies.

Serologic markers have been used to diagnose and categorize IBD. Perinuclear anti-neutrophil cytoplasmic antibodies (pANCA) have been linked predominantly to UC, and anti–*Saccharomyces cerevisiae* antibodies (ASCA) have been associated with CD (37–39). ASCA detect *S. cerevisiae* mannan, a cell wall carbohydrate that is common to most fungi. Thus, the specificity of ASCA for *Saccharomyces* is not clear, since other common fungi, including *Candida albicans*, have abundant mannan in their cell walls. Nevertheless, evidence of atypical immunologic activity against yeast (i.e., ASCA) in CD suggests that disease may be associated with an unusual exposure of the immune system to fungi.

Increases in both ASCA IgG and IgA are commonly observed in patients diagnosed with CD. The ASCA IgA and IgG positive rate is over 50% in patients with CD and less than 5% in patients with non-IBD colitis or healthy controls (40–42). In pediatric patients with CD, ASCA positivity has been associated with older children (>10 years), small bowel disease, and long-term risk of surgery (43, 44). In adults, ASCA has been linked to increases in disease severity, location, and age, with ASCA-positive patients more likely to have severe and complicated disease (45). A recent study of pediatric patients in Australia noted that ASCA positivity correlated with increases and decreases in several specific bacteria, further suggesting that ASCA may be associated with specific subtypes of disease and that this may be reflected in the microbiome as well (44).

Some studies suggest that appearance of ASCA may precede diagnosis and might contribute to a useful predictor of disease and disease risk. In 2005, Shoenfeld and coworkers reported following healthy soldiers enlisting in the Israeli armed services over a period of 3 years and identifying 32 people who developed CD and 10 who developed UC for whom serum samples had been archived before diagnosis (46). Among the CD patients, more than 30% were positive for ASCA before diagnosis, with the prevalence of the antibodies increasing up to 2 years before diagnosis. No evidence for ASCA in UC was observed. The numbers in the study were small, and a more recent US armed services study of 200 subjects who developed CD and 199 who developed UC, looking at seven antibody markers and over a thousand serum protein markers, came to a similar conclusion regarding ASCA (47). ASCA IgA was observed to be the most predictive marker of a future diagnosis of CD and was predictive as much as 5 years before diagnosis, although addition of further markers increased the sensitivity and specificity of the diagnosis. Again, in this study ASCA was not predictive of UC.

Biomarkers can be useful for identifying phenotypic differences between patients with IBD and might one day be helpful in making therapeutic choices. For example, clinical response to infliximab, an anti–TNF-α biologic therapy for both CD and UC, may be reduced in pANCA^+^ or pANCA^+^ASCA^–^ patients (48–50). Thus, specific antibodies may help to inform therapeutic choices and may provide insights into underlying mechanisms of disease subtypes. Further studies are required to better understand whether ASCA positivity, perhaps together with related genomics (i.e., *CARD9* genotype), might inform tailored courses of therapy. Still, it is important to clearly understand that while it seems highly likely that ASCA is generated in response to fungi, it is formally possible that the truly relevant antigen is bacterial or human. While humans and bacteria do not produce the long α-mannan polymers produced in abundance by yeasts, the linkages are not unrelated to structures found, for example, on human glycoproteins or in mycobacterial lipoarabinomannan. Further studies are required to better understand the source of this antibody activity.

## The fungal microbiome

Since genetic and serologic data suggest that alterations in immune responses to fungi might be important in determining the risk or severity of disease, the important questions arise of what fungi are in the gut and whether their presence or composition influences IBD. Large microbiome studies including the large-scale Integrative Human Microbiome Project (HMP1 and HMP2) rarely discuss fungal microbiota as a potentially meaningful component of the intestinal microbiome, while reporting characteristic increases in bacterial facultative anaerobes at the expense of obligate anaerobes in patients with IBD (51, 52). This is likely mostly because metagenomic sequencing attempts tend to report that less than 0.1% of the microbial genomes found in the gut are fungal (53, 54). It might follow that their influence is minimal. An important error in this presumption is that yeasts are up to 100-fold larger than bacteria, so that a smaller number of organisms represents an unexpectedly substantial fraction of the microbiome biomass. Also, as eukaryotes, they are likely to employ unique metabolic activities not found in prokaryotes such that their metabolic influences might be disproportionate to their abundance. Finally, intestinal fungal blooms, especially of *Candida* spp., have long been recognized as a common experience in humans, particularly as a consequence of taking oral antibiotics (55). In a mouse model of colitis, Iliev et al. reported that a rich mycobiome is present and that antifungal innate immunity is important for controlling inflammation (56), making the case for further studies on the topic.

Important progress has been made in recent years in defining fungi that are found in the human intestines (luminal and mucosal) and in beginning to think about how the immune system might interact with these organisms (2). Changes in the bacterial microbiota associated with IBD have been well documented, and many recent studies have now similarly documented that the fungal microbiota is altered in patients with IBD, although the nature of the reported changes varies. In an early study in this area, Ott et al. reported using metagenomic 18S ribosomal DNA–based denaturing gradient gel electrophoresis to observe a higher mean fungal diversity in colonic biopsy tissue samples from patients with CD in comparison with controls (57). A recent study of fecal samples from CD patients in the United Kingdom similarly reported greater fungal diversity associated with disease (58). In contrast, other recent ribosomal DNA sequencing–based studies of mucosa-associated microbes (59) or fecal samples (60) reported little or no change in fungal diversity in CD, although there were changes in apparent fungal burden and population structures. Somewhat more commonly, studies on fecal samples have tended to report reductions in fungal diversity associated with CD and UC (61, 62). Clearly there are ongoing challenges associated with characterizing the mycobiome.

Capturing a description of the fungal microbiome from stool or intestinal mucosal samples using sequencing approaches can be difficult. A large fraction of the fungi commonly identified in human and animal studies are likely to be environmental rather than truly commensal. Many are known to be plant pathogens (e.g., *Stenocarpella maydis*, *Fusarium* spp.) or common environmental molds (e.g., *Cladosporium* spp.), and many are well known to be prominent in and on food (e.g., *Saccharomyces* spp., *Debaryomyces* spp., *Penicillium* spp.). Lumping all of the environmental organisms together with the unknown “true” commensal organisms may obscure important disease-associated features of the mycobiome, although rigorously identifying the true commensal organisms is difficult. Treatment of mice with oral antifungal drugs reveals expansion of unlikely drug-resistant fungi such as *Wallemia sebi*, marking them as genuinely replicating in the gut (8, 63). Laboratory mice might serve as a poor model for fungal microbiome diversity and function, since one of the most striking features of the gut microbiome in recent studies with “rewilded” laboratory mice given or exposed to a wild microbiome is an enormous increase in the burden of fungi (64). Thus, typical laboratory mice are likely starved of fungal contact common in the wild. Future studies of the effects of oral antifungal drugs in humans might help clarify a broad set of organisms that are genuinely commensal.

Nevertheless, several fungi have stood out in different studies as being specifically altered in patients with IBD. Whether or how these commensal organisms might influence a person’s susceptibility to developing IBD or the severity of the disease is not fully understood.

### Candida.

A common, although not universal, finding has been an increase, statistically significant or trending, in the relative amount of *Candida* in the fecal mycobiome of patients with CD from diverse geographic locations (3, 58–62, 65). The named species is usually *C. albicans*, but *C. tropicalis* and *C. glabrata* have also been reported.

The GI tract of the majority of humans in Westernized societies is colonized by *C.* *albicans*, and this is the only fungus for which a large body of literature exists exploring its mechanisms of gut colonization (66). The broader composition of the microbiota influences *C.* *albicans* colonization and levels. Use of broad-spectrum antibiotics is recognized as a major risk factor for *Candida* overgrowth and risk of candidemia (67). In mice, germ-free animals are easily colonized, but many common laboratory mice are resistant to colonization. In humans, some positive and negative associations between *C.* *albicans* and various bacteria have been noted (65, 68–70), but the significance or underlying mechanisms of these relationships remain to be established.

Invasive infections with *Candida* species, especially *C. albicans*, are the most common cause of nosocomial fungal infections in patients in intensive care units, immunocompromised patients, or patients with dysfunctional epithelial barriers (66). Since *C.* *albicans* is a commensal organism and is not present in the environment like common molds, systemic candidiasis of humans originates from commensal sites. In fact, intra-abdominal candidiasis is a particular risk in the context of abdominal surgery (71). This may be especially relevant to CD patients, as a recent study in Slovakia found a significantly higher rate of extraintestinal *C.* *albicans* in CD patients undergoing surgery compared with a control group of cancer patients (72). A large clinical study of fecal microbiota transplant in UC found that higher *Candida* abundance before transplant (and reduced *Candida* levels after transplant) was associated with a favorable clinical response (69). These findings strengthen the association of fungal levels with disease activity and response to treatment, but their thoughtful design reveals gaps in cause-and-effect evidence and the specificity for *Candida*.

The best efforts to address causality and *Candida* in exacerbation of colitis are in mouse models, but observations to date are mixed. Notably, enteric *Candida* does not alone elicit colitis, and has not been tested in the context of mice bearing genetic susceptibility traits for colitis. In settings of impaired host fungal control mechanisms and induced mucosal injury, colitis is associated with blooms of multiple fungal taxa, among which *Candida* is prominent (56, 73). However, this may simply reflect its prevalence as a gut commensal, and the design did not permit assessing the specific role of *Candida* in disease activity. Conversely and paradoxically, selective suppression of *Candida* and certain other fungal taxa with chronic fungal antibiosis augments severity of model colitis (63). A series of studies by various groups document that oral administration of *Candida* can augment colitis elicited by dextran sulfate sodium (DSS), a commonly used agent that acts via acute metabolic epithelial injury (63, 74–80). Demonstrated direct virulence mechanisms are still emerging and include fungal chitin and β-mannosyltransferases. More consistently, the effects were linked to reciprocal ecologic effects on beneficial and colitogenic bacteria, suggesting that bacteria may serve as intermediaries of the host-*Candida* effect on colitis.

IgA antibodies targeting cell surface adhesins (enriched on hyphal structures) are largely protective against infection, in accord with the role of hyphae in virulence. Paradoxically, hyphal differentiation reduced fitness for gut colonization, a trait typically integrated with pathogenicity in other anatomic sites (81) but reflecting the recent appreciation that the yeast phenotypic state favors *Candida* gut commensalism (82, 83). Among gut mycobiome members, *Candida* is particularly antigenic for systemic IgG immunity, and anti-*Candida* IgG provides protection from systemic disseminated infection. Conversely, *Malassezia* was distinguished by its relatively reduced elicitation of systemic IgG to fungal surface antigens, but rather than immunologic neglect, this may reflect a concealment strategy by this fungal taxon related to its commensal lifestyle (84). Taken together, these observations suggest various contexts and distinct potential roles of *Candida* in models of colitis and host gut immunity, but more work is needed to understand whether these are truly relevant in the context of human IBD.

### Malassezia.

In a recent study, we analyzed the mycobiome by internal transcribed spacer (ITS) sequencing over 160 mucosal washing samples collected during colonoscopy of patients with CD and healthy controls. We observed a strong association of *Malassezia* spp. (*M.* *restricta* and *M.* *globosa*) with CD (85). Subsequently, Miller and coworkers observed a remarkable appearance of gut-derived *Malassezia* in pancreatic tumors, further supporting the notion that *Malassezia* is associated with dysbiosis-driven diseases (86). Also, Devkota and coworkers observed enrichment of *Malassezia* in surgical mucosal samples collected from CD, but not UC, patients (87). Earlier, Liguori et al. detected *Malassezia* in mucosal biopsies from CD patients, although the association with disease was not entirely clear (59). Interestingly, studies on fecal samples rarely report finding abundant *Malassezia* spp., suggesting that *Malassezia* spp. may be primarily residents of the intestinal mucosal, not the lumenal, microbiome.

*Malassezia* spp. are members of the Basidiomycota phyla of fungi, and while all other members of the Ustilaginomycotina subdivision are plant pathogens, *Malassezia* are commensal skin microbes found on nearly all warm-blooded animals. *Malassezia* have among the smallest of eukaryotic genomes, having only around 4000 genes. They grow mainly as yeasts, although some species can develop hyphae. An important feature of *Malassezia* genomes is the loss of key enzymes required for lipid metabolism, including fatty acid synthase, Δ-desaturase, and Δ-enoyl CoA isomerase (88). Therefore, they cannot produce fatty acids themselves and need lipids from the environment for growth. In the skin, they harvest lipids from sebum in hair follicles through secretion of a host of lipases and phospholipases. These enzymes can release unsaturated free fatty acids from sebum lipids including oleic acid and arachidonic acid that can be inflammatory. That *Malassezia* spp. might be important in intestinal inflammation is unexpected.

Among patients with CD, we found that the presence of *Malassezia* was positively linked to the presence of the *CARD9* IBD risk allele (85). We observed that CARD9 is important for inflammatory responses to *Malassezia* (89), and that the CARD9 IBD risk allele alters how immune cells respond to *Malassezia* (85). In a mouse model of colitis, *Malassezia* could exacerbate disease, and this effect required functional CARD9 (85). ASCA-positive sera cross-react with *Malassezia*, and further studies are required to better understand whether immune exposure to *Malassezia* could drive ASCA appearance.

### Other yeasts.

The ASCA seromarker of CD nominally targets the cell wall of *Saccharomyces cerevisiae*. It was early understood that the antigenic structure is widely shared among fungal taxa (90–93). Nonetheless, since *S.* *cerevisiae* is an established gut commensal (61, 94–96) (although some argue for dietary pass-through versus colonization) (96), several groups have tested the association of their fecal prevalence with human IBD. Such studies have consistently reported their selective *reduction* in IBD and CD (6, 59, 61). In mouse colitis models, a few groups have found that administration of *S.* *cerevisiae* or their polysaccharides ameliorates dextran sulfate colitis (97–99). However, the isolates were not of gut origin and, in design, reflected the search for *Saccharomyces* isolates with probiotic properties. With regard to such commensals, recent studies assessing uric acid dysmetabolism and its impact on colitis have associated this pathophysiology with *S.* *cerevisiae* in humans and mice (100, 101).

In other parenchymal sites, injured mucosa opens damaged epithelium to fungal colonization, resulting in delayed healing and chronic inflammatory phenotypes. Notable examples include *Malassezia* in the skin, and *Aspergillus* and other fungal taxa in the lung (102, 103). Poor recovery from mucosal injury is a common and clinically important feature in IBD. This has commonly been attributed to non-resolution of the primary inflammatory event and thus failure to direct the processes of wound closure and crypt regeneration (104–106). A recent study uncovered evidence for ulcer colonization by *Debaryomyces hansenii* as a contributor to ongoing mucosal inflammation in the gut (107). This yeast was selectively detected in colonic biopsy wounds and DSS-induced colonic injury in mice, and in human CD ulcers. *Debaryomyces* administration in mice slowed mucosal regeneration during colitis and injury, an effect that required the myeloid inflammatory network controlled by type I interferon and CCL5. This observation echoes precedents for opportunistic fungal colonization causing persistence of parenchymal injury in lung and skin lesions.

## Manipulating intestinal fungi

Observations of disease-associated differences in intestinal microbial populations in patients with IBD have prompted widespread interest in the idea that manipulating the gut microbiome may be beneficial. Still, it is not yet clear whether or when microbiota changes cause disease or are simply a result of disease. Approaches to manipulating the microbiome include fecal microbiota transplantation (FMT) and the use of medications (i.e., antibiotics), probiotics, and prebiotics.

### Fecal microbiota transplantation.

FMT has proven to be an astoundingly effective treatment against antibiotic-resistant *Clostridioides difficile* infection, indicating that it can be an effective way to “reset” the intestinal microbiota to be resistant against infection. But can it be an effective treatment for IBD? Reports from small randomized, controlled clinical trials employing FMT to treat patients with IBD are mixed (108–111). In patients with UC, one trial showed no benefit, while three have suggested varying degrees of efficacy. However, the studies used different methods of FMT administration (nasoduodenal infusion, enema, colonoscopy), dose frequency (daily or weekly), and formulation of the FMT product (fresh or frozen, single donor or pooled donors). FMT thus remains an experimental treatment requiring further study. A key challenge is defining the patients for whom FMT might be warranted and defining the technical parameters that make FMT effective. While most interest in this space focuses on characterizing bacterial populations, perhaps the fungal component is a key.

Leonardi et al. recently reported an investigation into whether the presence of specific fungi detected by ITS sequencing before or after FMT might identify UC patients who respond to treatment (69). They reanalyzed samples from a previous FMT study of UC (109, 112) that had reported bacterial and metabolite profiles of samples. The previous study had not found any single factor that seemed to associate with response to treatment, finding generally that response was associated with greater bacterial diversity, increases in *Eubacterium hallii* and *Roseburia inulivorans*, and increases in levels of short-chain fatty acid biosynthesis and secondary bile acids. Patients who did not respond to the FMT were characterized by having more *Fusobacterium gonidiaformans*, *Sutterella wadsworthensis*, and *Escherichia* spp. and increased levels of heme and lipopolysaccharide biosynthesis. Leonardi et al. found further that high levels of *C. albicans* in patient samples prior to FMT were associated with a good clinical response and that this response was associated with a decrease in *C.* *albicans*. How this ultimately relates to the net environment that permits FMT to be effective will require further mechanistic study.

### Antibiotics and probiotics.

Data are similarly mixed for the use of antibiotics to treat patients with IBD. While some meta-analyses suggest that antibiotics might be helpful in some patients (113), the data are inconsistent, and it is not clear whether the risks of antibiotic use outweigh the putative (mostly short-term) benefits. Would antifungal treatment be helpful in any of these patients? A small case series suggested clinical benefit to underlying CD in 4 of 6 patients treated with the antifungal agent itraconazole for histoplasmosis (114). In another small study directly examining use of fluconazole for postoperative recurrence of CD, drug treatment did not affect recurrence. After surgery, *C.* *albicans* colonization, biomarkers of *C.* *albicans* pathogenic transition, and antifungal antibodies (against *Candida* and generic fungal polysaccharides) significantly declined regardless of prior fluconazole treatment (115). It seems that larger studies with refined design will be necessary to unravel these observations.

Uses of pro- and prebiotics to influence the makeup of the gut microbiome for benefit are popular among patients, but there are insufficient data supporting their efficacy for CD. In UC, several studies suggest that bacterial probiotics might provide modest benefit in preventing disease recurrence, although given the variety of probiotics and wide variations in patient profiles, the data are difficult to interpret (116). The effects of probiotics on fungal microbiota are not known.

The most widely used probiotic yeast is *Saccharomyces boulardii*, a yeast closely related to baker’s yeast that is used for prophylaxis and treatment of infectious antibiotic-associated diarrheal diseases and management of preterm infants (117–120). Reports of utility of *S.* *boulardii* in IBD have been around a long time, but the literature today is mixed and ultimately inconclusive (121). Animal models have been used to observe the efficacy of *S.* *boulardii* in colitis, with some movement toward understanding potential mechanisms of action. In a notable recent study, amelioration of DSS-induced colitis by *S.* *boulardii* required their integration with gut Enterobacteriaceae (75). That is, protection by *S.* *boulardii* selectively required microbiome presence of this bacterial genus, owing at least in part to facilitation of fungal colonization. Similarly, but paradoxically, the pathogenic effects of *Candida* spp. were accordingly augmented by Enterobacteriaceae (see “The fungal microbiome” section above). The mechanism by which these bacteria promote fungal colonization is yet undetermined. An alternative hypothesis arising from laboratory models suggests that *S.* *boulardii* may promote epithelial barrier health, owing to its augmentation of E-cadherin–dependent inter-epithelial junction formation and barrier integrity (122, 123). A better understanding of the mechanisms of action underlying these protective effects could help better identify the subset of patients who might benefit from probiotic treatments.

## Toward understanding fungi in the total microbiome

Both conceptual thinking and experimental evidence have established the gut microbiome as a key component of disease risk, phenotype, and activity in IBD. This Review distills the current but still early understanding of the fungal community in this disease biology. How can we deepen and accelerate this understanding?

An important precedent to guide us are the extensive studies of the impact of the soil microbiome on plant productivity and disease mitigation. Given the salience of fungal pathogens in plant disease, an interkingdom perspective was adopted earlier in study of the soil microbiome using conventional microbiology and accelerated with the advent of high-dimensional genomic microbial and fungal taxonomics and network bioinformatics. At present, there are deep awareness and robust data sets assessing the ecologic interplay of bacterial and fungal members of the soil microbiome, and their roles in plant phenotypes. This includes interkingdom ecologic networks and redundant taxonomic ensembles mediating key properties required for plant productivity, such as carbon sequestration and nitrification, and the identity of proximate plant pathogens and the organisms and products that act to suppress them (124, 125).

Two examples of plant pathogens and suppression of their pathogenicity illuminate approaches to gut mycobiome studies that lie ahead (126, 127). In the common “take-all” disease, a fungal taxon, *Gaeumannomyces tritici*, infects root tissue of wheat and barley, progressing to invasive blockage of plant water-conductive tissue and resultant loss of plant yield and viability. Some soils are strongly suppressive of disease, such that mixing 1% suppressive soil averted disease in susceptible soils. Microbiome compositional analysis associated this trait with a variety of *Pseudomonas*, and metabolomics and mechanistic studies linked this trait to 2,4-diacetylphloroglucinol, a secreted antifungal metabolite of these pseudomonads. In a second example, certain anastomosis groups (AGs) of the basidiomycete *Rhizoctonia solani* target a diversity of plants. Suppression of this pathogen group is a common soil trait, and conventional culture methods and sequence-based association studies uncovered candidate bacteria with this suppressive property. However, with the advent of fungal sequence-based microbiome analysis, fungal taxa were also associated with a suppressive phenotype, including *Bionectria*, *Xylaria*, and *Eutypa* in AG-8 (in wheat), and *Trichoderma* in AG-2-2 (in sugar beets). Mechanistic studies have established several properties by which *Trichoderma* promotes protection, including chitinase, proteases, β-1,3-glucanase, detoxification of host-targeting pathogenic products, and ecologic competition with soil fungal pathogens (128).

While the concept of pathogenic gut microbiome members is widely held for IBD, the definition of proximate ecologic pathogens is less developed than in the soil paradigm. Some bacterial and fungal candidates and their mechanisms are emerging, including the examples of *Candida* and *Malassezia* discussed above. Similarly, discovering and characterizing disease-suppressive gut microbiomes is a powerful analytic tool for IBD microbiome pathogenesis, and also an appealing starting point for microbiome-targeted therapeutics. The search for such protective communities has lagged behind the search for pathogen-bearing microbiomes in mouse models and human association studies. From this perspective, the effectiveness of FMT in human UC and the variability of donor FMT effectiveness is a promising tool.

Study design and analytic strategies to assess the fungal microbiome have matured enough for ready adoption by translational investigators. These include standardized approaches for efficient release of fungal DNA from biologic specimens; kingdom-wide library production and robust ITS databases for molecular taxonomic annotation; and joint analysis of bacterial and fungal microbiomes to deconvolute their primary versus dependent contributions to host biologic states. Looking ahead, adoption of bacterial metatranscriptomic (51, 52) and molecular replication (129) methods will provide metrics to better define fungal biologic activity in situ, but this will require a substantial scientific community effort to assemble comprehensive fungal metagenomic databases (130, 131). Recent bacterial microbiome investigation has been propelled by the development and sharing of large-scale gut isolate culture libraries (132). Development of corresponding fungal libraries will improve prospects to more robustly screen candidate organisms for causal host biologic activities in mouse models and support proof-of-concept probiotic applications. However, such studies must also overcome barriers to targeted fungal colonization in mice.

Pathway-based genetic and cell biology methods (10) will be important to fastidiously uncover and validate candidate fungal taxa and their products. In the discovery phase, these methods must be integrated with recent refinements in fungal metagenomics and structure-informed metabolomics. Finally, an impressive recent advance was the large-scale culture and multi-omics analysis of gut-derived bacteria, which provides a powerful tool to associate and deconvolute microbiome members with luminal bioactive products (132). Development of such a research resource for the fungal gut microbiome would accelerate insights on fungal roles in host-microbiome relationships such as IBD.

## Conclusion

We are cautiously enthusiastic about the potential for studies on the fungal microbiota to influence our understanding and treatment of IBD. Abundant high-quality data are available to support the hypothesis that modulation of the fungal microbiota may be important in the development and severity of IBD in some patients. Ecologically, this includes both direct host-fungal interactions, and those mediated via fungal-bacterial ecologic events. Alternative explanations and hypotheses related to these data remain possible. We look forward to the incorporation of available tools for fungal analysis by basic and translational investigators to advance and refine the understanding of fungal participation in IBD pathogenesis and disease activity.

## Version 1 03/01/2022

Electronic publication
